# Apremilast for acquired reactive perforating collagenosis with metabolic and infectious comorbidities: a case report

**DOI:** 10.3389/fimmu.2026.1671943

**Published:** 2026-06-30

**Authors:** Fujun Huang, Lei Tang, Mengya Huang, Qiang Fu, Xun Zhou, Zhong Zhang, Mingdan Zhao

**Affiliations:** 1 Department of Cosmetic Dermatology, Chongqing Traditional Chinese Medicine Hospital, Chongqing, China; 2 Liaoning University of Traditional Chinese Medicine, Shenyang, Liaoning, China; 3 Chengdu University of Traditional Chinese Medicine, Chengdu, Sichuan, China; 4 Department of Dermatology, Chongqing Traditional Chinese Medicine Hospital, Chongqing, China

**Keywords:** acquired reactive perforating collagenosis, apremilast, case report, comorbidities, treatment

## Abstract

Acquired reactive perforating collagenosis (ARPC) is a rare disorder characterized by transepidermal expulsion of altered collagen, for which no standardized treatment is available. We present a case of a 67-year-old man with ARPC and significant comorbidities, including type 2 diabetes mellitus (T2DM), chronic hepatitis B (CHB), and impaired pulmonary function, who achieved rapid and substantial improvement in cutaneous lesions and pruritus after treatment with oral apremilast therapy. Notably, his comorbid conditions remained stable, and the clinical improvement persisted even after a seven-month drug-free follow-up period. This case highlights apremilast as a potentially effective and safe treatment option for ARPC, especially in patients unsuitable for conventional immunosuppressive therapy.

## 1 Introduction

Acquired reactive perforating collagenosis (ARPC) is a subtype of perforating dermatoses, pathologically characterized by transepidermal expulsion of altered collagen bundles and clinically characterized by severe pruritus (1, 2). In 1994, Faver et al. initially proposed the diagnostic criteria for ARPC: ① histopathologic findings of expulsion of necrotic basophilic collagen bundles into a cup-shaped epidermal depression, ② clinical lesions of umbilicated papules or nodules with a central, adherent keratotic plug, and ③ onset of the lesions at an age of ≥18 years old (3). Current treatment options for ARPC include combination pharmacotherapy (most commonly oral antihistamines and topical corticosteroids), phototherapy, dupilumab, Janus kinase (JAK) inhibitors (e.g., tofacitinib, abrocitinib, baricitinib), and allopurinol (2, 4–6). A follow-up conducted for six months after treatment discontinuation showed a recurrence rate of 16.7% (7). In this report, we describe a patient with ARPC and multiple comorbidities who achieved substantial and sustained clinical improvement after treatment with apremilast.

## 2 Case report

**Figure 1:**
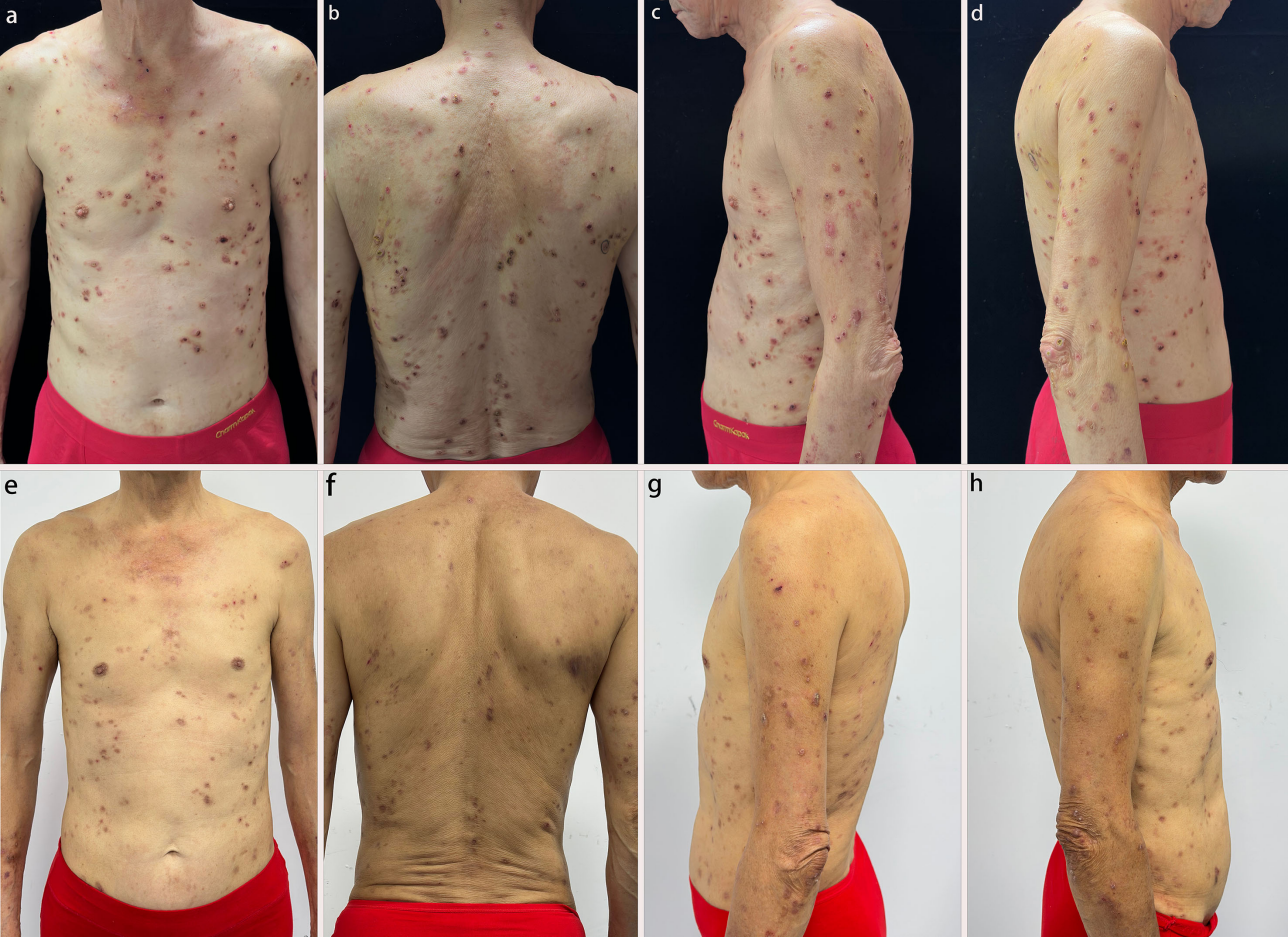
Clinical manifestations of ARPC before and after apremilast treatment. **(a–d)** Baseline clinical presentation showing multiple variably sized umbilicated erythematous papules and nodules with central adherent keratotic plugs distributed on the trunk and upper extremities. **(e–h)** Marked clinical improvement after oral apremilast treatment, with substantial regression and flattening of the lesions, reduced erythema, and residual post-inflammatory hyperpigmentation.

**Figure 2:**
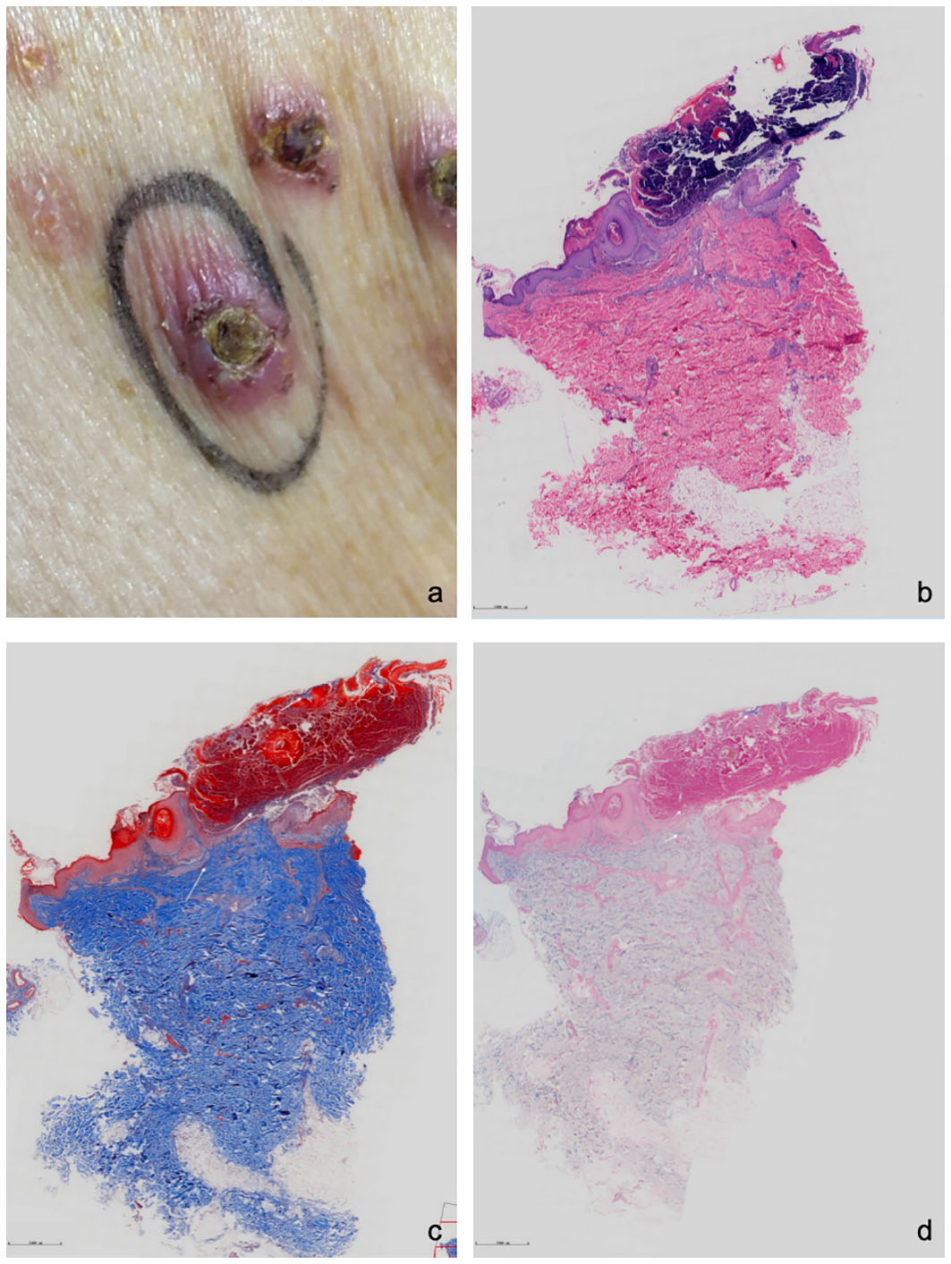
Representative clinical and histopathological features of ARPC. **(a)** Close-up clinical image showing multiple umbilicated erythematous papules and nodules with central adherent keratotic plugs; the circled lesion indicates the biopsy site. **(b)** Hematoxylin and eosin staining showing epidermal invagination with a central keratotic plug and transepidermal elimination of dermal material. **(c, d)** Masson’s trichrome staining and Victoria blue staining showing transepidermal elimination of degenerated collagen fibers from the dermis into the epidermis, indicated by white arrows.

In September 2024, a patient initially diagnosed with eczema was admitted to the hospital due to an inadequate response to repeated therapy with systemic antihistamines combined with topical corticosteroids. The patient manifested the following clinical characteristics: ① baseline: This case involved a 67-year-old man; ② history of present illness: At six months before admission, the patient developed generalized pruritus without identifiable precipitating factors, followed by umbilicated papules or nodules in variable sizes with a central, adherent keratotic plug (**Figures 1a–d** and **Figure 2a**). An initial diagnosis of “eczema” was made at a local hospital, but treatment with oral antihistamines and topical corticosteroids failed to achieve symptomatic relief; ③ past medical history: There was a 6-month history of type 2 diabetes mellitus (T2DM), chronic hepatitis B (CHB), and heterogeneous ground-glass opacities in bilateral lower lobes revealed by chest computed tomography (CT), suggestive of hypoventilation; and ④ negative family history.

Upon admission, a series of laboratory tests were performed, including complete blood count (CBC), urinalysis, stool examination, C-reactive protein (CRP), liver function tests (LFTs), renal function tests, lipid profile, fasting blood glucose (FBG), hemoglobin A1c (HbA1c), electrolytes, hepatitis B virus (HBV) serological markers, hepatitis C virus antibodies, T-SPOT.TB test, Treponema pallidum antibody (TP-Ab), human immunodeficiency virus (HIV) screening, desmoglein 1 antibody (Dsg1), Dsg3, serum immunoglobulins, complement C3, complement C4, antinuclear antibody (ANA), and anti-extractable nuclear antigen (ENA) antibodies. Most results were within normal limits, except for FBG (9.56 mmol/L), HbA1c (7.8%), white blood cell count (WBC) (11.79 × 10^9^/L), neutrophil percentage (NEUT%) (80.1%), absolute eosinophil count (AEC) (0.74 × 10^9^/L), CRP (11.03 mg/L), hepatitis B surface antigen (HBsAg) (16.275 IU/mL), hepatitis B e antibody (HBeAb) (>6.000 IU/mL), hepatitis B core antibody (HBcAb) (>80.000 PEI U/mL), and HBV DNA (2.118 × 10³ IU/mL). Histopathology revealed hyperkeratosis, epidermal invagination, dermal perivascular lymphocytic infiltrates (H&E staining; **Figure 2b**), vertically extruded degenerated collagen fibers (Masson’s trichrome staining; **Figure 2c**), and fragmented elastic fibers (Victoria blue; **Figure 2d**). ARPC was diagnosed based on the clinical findings, histopathologic findings, and patient’s age made (3).

**Figure 3:**
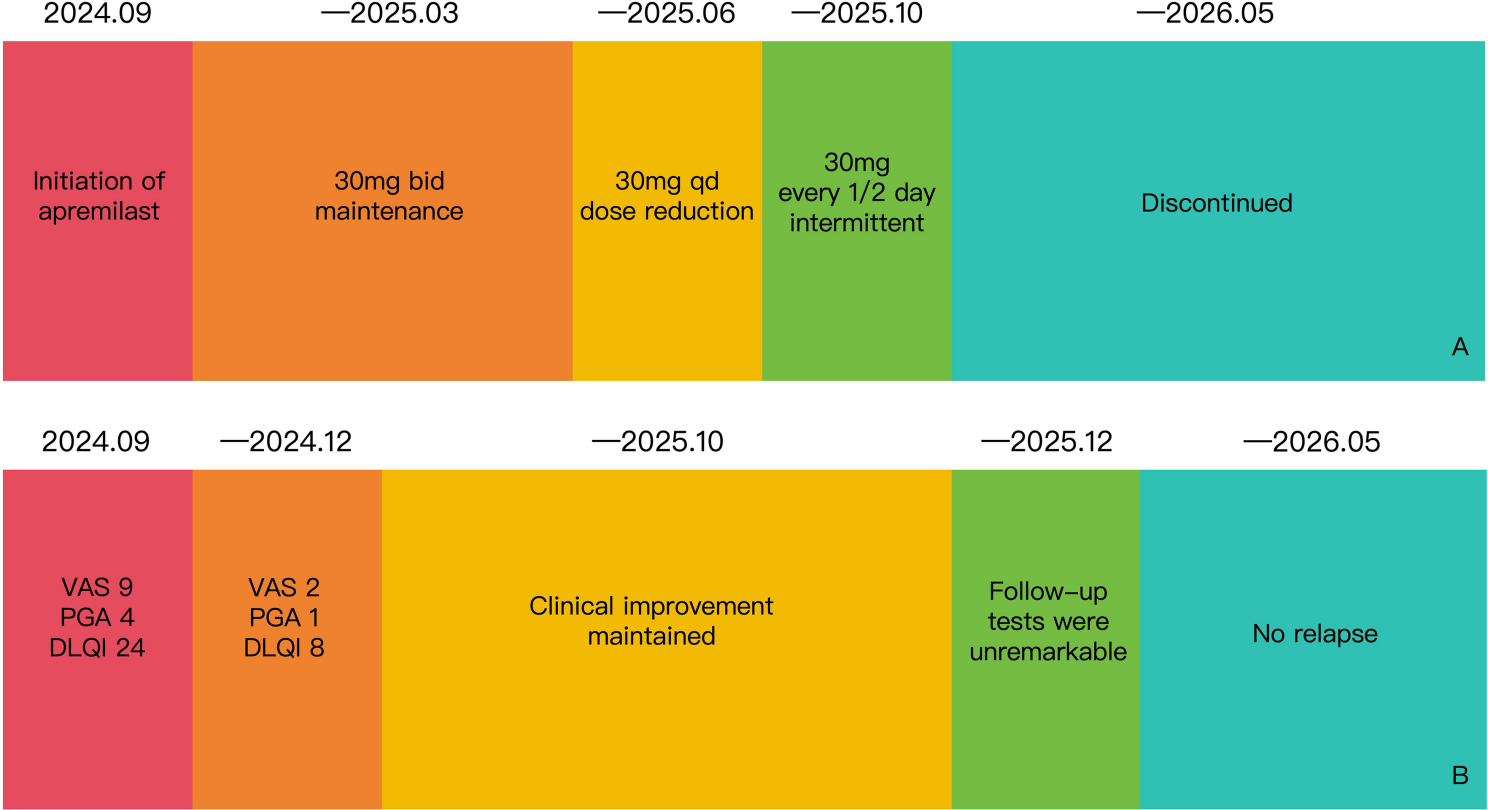
Treatment timeline and clinical response during apremilast therapy. **(A)** Timeline of oral apremilast treatment, including treatment initiation. **(B)** Timeline of clinical response and follow-up outcomes.

Given the following considerations, dupilumab or apremilast was considered for the patient: ① The patient presented with severe clinical manifestations (Visual Analog Scale [VAS] score of 9, Physician’s Global Assessment [PGA] score of 4, and Dermatology Life Quality Index [DLQI] score of 24) and demonstrated an inadequate response to long-term outpatient treatment with oral antihistamines and topical corticosteroids. ② The patient had contraindications to immunosuppressants and JAK inhibitors, including poorly controlled T2DM, active CHB, and impaired pulmonary function (8, 9). ③ A history of suboptimal treatment adherence posed a significant challenge to phototherapy, and the required frequency of administration typically at three sessions per week could increase the risk of treatment discontinuation (10). Moreover, phototherapy is associated with an increased risk of cutaneous malignancy (11). ④ Given the presence of active chronic hepatitis B in the patient, the potential hepatotoxicity of allopurinol raised concerns about the exacerbation of pre-existing liver dysfunction or the induction of liver function abnormalities (12). ⑤ For patients with diabetes and suboptimal glycemic control, systemic retinoid therapy may exacerbate dysregulation of glucose and lipid metabolism (13–15). Considering the severity of the disease and the existence of multiple comorbidities, as well as the evidence from previous studies conducted by McMichael et al. and Liu et al., dupilumab or apremilast was considered a potential treatment option (6, 16). The final therapeutic regimen was determined after comprehensive consideration of the drug formulation, route of administration, and patient’s economic circumstances. Apremilast was initiated using the standard titration regimen, reaching a dose of 30 mg twice daily by Day 7. The specific treatment regimen and clinical response are detailed in **Figure 3**.

At 12 weeks, lesions had regressed by >75% with flattened papules and resolution of erythema (**Figures 1e–h**). The severity of symptoms (VAS: 9→2; PGA: 4→1) and quality of life (DLQI: 24→8) improved significantly. No hepatic, renal, glycemic, or infectious complications were observed after 12 weeks of oral apremilast therapy. Notably, following the diagnosis of ARPC in September 2024, the patient received oral apremilast therapy intermittently until October 2025. Laboratory tests including CBC, CRP, LFTs, renal function tests, lipid profile, FBG, and HbA1c were performed in December 2025, revealing that FBG (7.88 mmol/L) and HbA1c (8.0%) were elevated, while all other parameters remained within normal limits. Telephone follow-ups in March and May 2026 showed complete resolution of his cutaneous lesions and pruritus after discontinuation of oral apremilast therapy, with no evidence of recurrence.

## 3 Discussion

Perforating dermatoses are a group of skin diseases characterized by transepidermal elimination of dermal or follicular material. Their main clinical and histopathological features are summarized in **Table 1** (3, 17–22). Although the exact prevalence and incidence of RPC/ARPC remain unknown, the condition is generally considered rare. In contrast, acquired perforating dermatosis, a broader category, has an estimated incidence of approximately 2.53 cases per 100,000 persons per year, with acquired reactive perforating collagenosis reported as one of its most common subtypes (19). RPC, was first reported by Mehregan et al. in 1967 (1). It can be classified into two types, namely hereditary and acquired, based on etiology. The former usually occurs in children and is presumed to follow a pattern of either autosomal recessive or autosomal dominant inheritance. The latter, appearing after 18 years of age, is termed ARPC and is associated with systemic diseases, especially diabetes mellitus (type 1 or 2) or chronic kidney disease, amid approximately 90% of patients (3, 23). The pathogenesis of ARPC primarily involves the transepidermal expulsion of altered collagen from the dermis, although the exact etiology and underlying mechanisms remain unclear. Mehregan et al. suggested that superficial microtrauma resulting from itching and scratching may induce collagen degeneration or apoptosis in the dermal papillae, thereby contributing to the onset of ARPC (1). This view is supported by the fact that clinical symptoms of ARPC can be alleviated after pruritus is controlled.

**Table 1:** Classification, clinical features, and histopathological findings of perforating dermatoses.

Disease	Clinical features	Histopathological features	Eliminated material
PCR/APCR	Pruritic umbilicated papules or nodules with a central keratotic plug; often occurs on extensor surfaces; acquired cases usually occur in adults and may be associated with diabetes mellitus or chronic kidney disease.	Cup-shaped epidermal invagination with a keratin plug; vertically oriented degenerated collagen fibers are extruded through the epidermis.	Collagen
Acquired perforating dermatosis	Adult-onset pruritic hyperkeratotic papules or nodules; commonly associated with diabetes mellitus, chronic kidney disease, or dialysis.	Cup-shaped epidermal invagination with a central keratotic plug and transepidermal elimination of dermal material, most commonly degenerated collagen, but sometimes elastic fibers, keratin, or mixed material. Follicular involvement may be present.	Collagen, elastic fibers, keratin, or mixed material
Elastosis perforans serpiginosa	Small keratotic papules arranged in annular, linear, or serpiginous patterns; may be idiopathic or associated with connective tissue disorders.	Epidermal invagination with transepidermal elimination of abnormal elastic fibers; elastic fibers are well demonstrated by elastic stains.	Elastic fibers
Kyrle disease	Hyperkeratotic papules or nodules with a central keratotic plug, usually on the lower extremities; often associated with diabetes mellitus or renal disease.	Keratotic plug with abnormal keratinization and transepidermal elimination of keratinous material; collagen and elastic fiber elimination is usually absent.	Keratin
Perforating folliculitis	Follicular papules, often on the extremities or buttocks; may be pruritic and may occur in patients with renal disease.	Dilated or disrupted follicular infundibulum containing keratin, inflammatory debris, and sometimes altered collagen or elastic fibers.	Follicular contents, keratin, ± collagen or elastic fibers

Therefore, some scholars have investigated the pathogenesis of ARPC with a focus on pruritus. Kawakami et al. found that µ-opioid receptors were overexpressed in the cytoplasm of upper and middle epidermal keratinocytes in ARPC lesions. Furthermore, it was demonstrated through reverse transcription quantitative polymerase chain reaction (RT-qPCR) and immunofluorescence analysis that interleukin-31 (IL-31) was also overexpressed in these lesions (24). his finding was supported by the successful treatment of a patient with ARPC using nemolizumab, an inhibitor of IL-31 receptor A (IL-31RA) (25). Meanwhile, several case reports have described the successful treatment of ARPC with dupilumab (26). Liu et al. explored the immunological mechanisms of ARPC and concluded that T helper type 2 (Th2) cytokines, mainly IL-4 and IL-13, are involved in the pathogenesis of ARPC. They further hypothesized that collagen degeneration induced by scratching or microangiopathy may impair metabolite clearance, thereby triggering type 2 inflammation and promoting ARPC progression (26). Given that the Th2 cytokines IL-4, IL-13 and IL-31, which are associated with the development of ARPC, signal through the JAK signal transducer and activator of transcription (JAK-STAT) pathway, and that this pathway is also involved in peripheral neuromodulation related to pruritus transmission, the successful treatment of ARPC with JAK inhibitors further supports this pathogenic mechanism (4–6, 27). This complex pathogenesis may also explain the limited efficacy of conventional antipruritic and anti-inflammatory treatments in our patient. Oral antihistamines mainly target histamine-mediated pruritus, whereas pruritus in ARPC appears to involve multiple non-histaminergic pathways, including IL-31, Th2 cytokines, JAK-STAT, and opioid receptor-related signaling pathways. Similarly, topical corticosteroids may reduce superficial inflammation but are unlikely to sufficiently reverse collagen degeneration, transepidermal elimination of altered collagen, or the itch-scratch cycle that perpetuates lesion formation. In addition, the presence of hyperkeratotic plugs and underlying systemic conditions, such as diabetes mellitus or chronic kidney disease, may further compromise the therapeutic response to such treatments.

Apremilast, a phosphodiesterase 4 (PDE4) inhibitor, modulates inflammation through two main mechanisms (28): ① activation of the cAMP-protein kinase A (PKA) pathway, which increases intracellular cAMP levels to suppress the NF-κB-mediated production of pro-inflammatory cytokines, including TNF-α, IL-17, and IL-22, while simultaneously promoting the release of anti-inflammatory IL-10; and ② broad immunomodulatory effects on keratinocytes, T lymphocytes, and dendritic cells, consistent with the multifactorial pathophysiology of ARPC.

## 4 Conclusion

In the present case, apremilast was selected because conventional immunosuppressive therapies were considered unsuitable in the context of T2DM, active CHB, and impaired pulmonary function. The patient achieved rapid and marked improvement in both pruritus and cutaneous lesions, without evidence of hepatic, renal, glycemic, or infectious complications during treatment with apremilast. Importantly, the clinical benefit was sustained after treatment discontinuation. This case suggests that apremilast may represent a useful treatment option for ARPC, particularly in patients for whom immunosuppressants, JAK inhibitors, phototherapy, or retinoids are contraindicated or considered less suitable. Its oral administration, favorable long-term safety profile, and reported low risk of HBV reactivation may be particularly relevant in such settings (29). However, this is a single case report; therefore, a causal relationship cannot be established, and larger studies are required to further validate these findings. Apremilast has also been approved for pediatric use, which may further support future exploration of its therapeutic potential in congenital forms of RPC (30). As targeted small-molecule therapies continue to evolve, the accumulation of additional clinical evidence may help guide selection of therapies for patients with ARPC and complex comorbidities.

## References

[1] Mehregan AH . Reactive perforating collagenosis. Arch Dermatol. (1967) 96:277. doi: 10.1001/archderm.1967.01610030055009 4166922

[2] Junghetu MA Tutunaru CV Ianoşi SL Georgescu CV Orzan OA . Acquired reactive perforating collagenosis—a rare entity occurring within common disorders: a systematic review and our personal experience. J Clin Med. (2026) 15:391. doi: 10.3390/jcm15010391 41517640 PMC12787290

[3] Faver IR Daoud MS Daniel Su WP . Acquired reactive perforating collagenosis. J Am Acad Dermatol. (1994) 30:575–80. doi: 10.1016/S0190-9622(94)70065-6 8157784

[4] Yuan R Zhou G Liu H . Tofacitinib for treatment of acquired reactive perforating collagenosis. JAMA Dermatol. (2025) 161:446. doi: 10.1001/jamadermatol.2024.6280 40009392

[5] Zheng J Ding Y Chen Y Shi Y Gao Y . Effectiveness of baricitinib in acquired reactive perforating collagenosis: a case report. Front Immunol. (2024) 15. doi: 10.3389/fimmu.2024.1388274 39076971 PMC11284048

[6] Liu B Fu M Zheng K Gao M Zou X He N . The treatment for acquired reactive perforating collagenosis with abrocitinib: a case report. JAAD Case Rep. (2025) 64:119–21. doi: 10.1016/j.jdcr.2025.07.005 40948681 PMC12423700

[7] Zhang Q Chang J . Acquired reactive perforating collagenosis: clinicopathologic analysis of 12 cases. Clin Cosmet Investig Dermatol. (2025) 18:859–65. doi: 10.2147/CCID.S502121 40225310 PMC11992978

[8] Virtanen A Spinelli FR Telliez JB O’Shea JJ Silvennoinen O Gadina M . JAK inhibitor selectivity: new opportunities, better drugs? Nat Rev Rheumatol. (2024) 20:649–65. doi: 10.1038/s41584-024-01153-1 39251770

[9] Winthrop KL Cohen SB . Oral surveillance and JAK inhibitor safety: the theory of relativity. Nat Rev Rheumatol. (2022) 18:301–4. doi: 10.1038/s41584-022-00767-7 35318462 PMC8939241

[10] Gao L Gu L Chen Z Cao S . Doxycycline combined with NB-UVB phototherapy for acquired reactive perforating collagenosis. Ther Clin Risk Manag. (2020) 16:917–21. doi: 10.2147/TCRM.S271058 33061396 PMC7522401

[11] Fowler JC Sood RK Coltart G Lai C Nadarajah N Holloway JW . Mutation burden of narrowband ultraviolet B phototherapy (NB-UVB) in human skin: relevance to NB-UVB lifetime exposures and skin cancer surveillance. Br J Dermatol. (2025) 193:718–28. doi: 10.1093/bjd/ljaf173 40317189 PMC12448954

[12] Wu H Wang C . Case report and analysis of allopurinol-caused ADR/ADE in 60 patients. Chin J New Drugs. (2013) 22:2215–8.

[13] Sachdev SS Jamil A Gunabalasingam P Safdar NA . The effects of acitretin on insulin resistance, glucose metabolism, and lipid levels in patients with psoriasis. Indian J Dermatol. (2022) 67:349–54. doi: 10.4103/ijd.ijd_328_21 36578705 PMC9792046

[14] Hartmann D Forgo I Dubach UC Hennes U . Effect of acitretin on the response to an intravenous glucose tolerance test in healthy volunteers. Eur J Clin Pharmacol. (1992) 42:523–8. doi: 10.1007/BF00314862 1535045

[15] Qian H Kuang Y Su J Chen M Chen X Lv C . Reductive effect of acitretin on blood glucose levels in Chinese patients with psoriasis. Front Med (Lausanne). (2021) 8. doi: 10.3389/fmed.2021.764216 34977070 PMC8716687

[16] McMichael J Stoff BK . Treatment of a perforating dermatosis with apremilast. JAAD Case Rep. (2021) 16:155–7. doi: 10.1016/j.jdcr.2021.08.031 34632026 PMC8488176

[17] Rice AS Zedek D . Kyrle Disease. (2026). 30422481

[18] Mullins TB Sickinger M Zito PM . Reactive Perforating Collagenosis. (2026). 29083792

[19] Harbaoui S Litaiem N . Acquired Perforating Dermatosis. (2026). 30969537

[20] Edek YC Aypek Y Öğüt B Erdem Ö Adışen E . Acquired perforating dermatosis: clinical and histopathological analysis of 95 patients from one center. Dermatol Pract Concept. (2024) 14:e2024100. doi: 10.5826/dpc.1402a100 38810077 PMC11135951

[21] Lv H Li M Cheng R . Novel small‐insertion mutation in the LIPH gene in a patient with autosomal recessive woolly hair/hypotrichosis. J Dermatol. (2020) 47:1445–9. doi: 10.1111/1346-8138.15581 32901930

[22] Patterson JW . The perforating disorders. J Am Acad Dermatol. (1984) 10:561–81. doi: 10.1016/S0190-9622(84)80259-5 6371074

[23] Mullins TB Sickinger M Zito PM . Reactive Perforating Collagenosis. StatPearls. (2024). 29083792

[24] Kawakami T Ikeda T Yokoyama K Dong Y . μ-opioid receptor overexpression in acquired reactive perforating collagenosis associated with IL-31. J Dermatol Sci. (2023) 110:69–71. doi: 10.1016/j.jdermsci.2023.04.003 37120414

[25] Yamada K Baba A Kanekura T . A giant variant of acquired reactive perforating collagenosis successfully treated with nemolizumab. J Eur Acad Dermatol Venereol. (2024) 38:e750–e752. doi: 10.1111/jdv.19864 38358063

[26] Liu B Wu Y Wu X Zhong X Xue R Zhang Z . Dupilumab improve acquired reactive perforating collagenosis characterized by type 2 inflammation. Front Immunol. (2023) 14. doi: 10.3389/fimmu.2023.1240262 37638036 PMC10449391

[27] Huang I-H Chung W-H Wu P-C Chen C-B . JAK–STAT signaling pathway in the pathogenesis of atopic dermatitis: an updated review. Front Immunol. (2022) 13. doi: 10.3389/fimmu.2022.1068260 36569854 PMC9773077

[28] Nassim D Alajmi A Jfri A Pehr K . Apremilast in dermatology: a review of literature. Dermatol Ther. (2020) 33:e14261. doi: 10.1111/dth.14261 32876993

[29] Piaserico S Messina F Russo FP . Managing psoriasis in patients with HBV or HCV infection: practical considerations. Am J Clin Dermatol. (2019) 20:829–45. doi: 10.1007/s40257-019-00457-3 31222626

[30] Blair HA . Apremilast: first pediatric approval. Pediatr Drugs. (2025) 27:119–24. doi: 10.1007/s40272-024-00668-0 39576565

